# Spatiomolecular Characterization of Dopamine D2 Receptors Cells in the Mouse External Globus Pallidus

**DOI:** 10.2174/1570159X21666230720121027

**Published:** 2023-07-31

**Authors:** Julie Espallergues, Jihane Boubaker-Vitre, Audrey Mignon, Maelle Avrillon, Morgane Le Bon-Jego, Jerome Baufreton, Emmanuel Valjent

**Affiliations:** 1 IGF, University Montpellier, CNRS, Inserm, F-34094 Montpellier, France;; 2 University Bordeaux, CNRS, IMN, UMR 5293, F-33000 Bordeaux, France

**Keywords:** Dopamine, pallidostriatal, globus pallidus, cholinergic neurons, mouse, D2Rs

## Abstract

The external globus pallidus (GPe) is part of the basal ganglia circuit and plays a key role in controlling the actions. Although, many evidence indicate that dopamine through its activation of dopamine D2 receptors (D2Rs) modulates the GPe neuronal activity, the precise spatiomolecular characterization of cell populations expressing D2Rs in the mouse GPe is still lacking. By combining single molecule *in situ* hybridization, cell type-specific imaging analyses, and electrophysiology slice recordings, we found that GPe D2R cells are neurons preferentially localized in the caudal portion of GPe. These neurons comprising pallido-striatal, pallido-nigral, and pallido-cortical neurons segregate into two distinct populations displaying molecular and electrophysiological features of GPe GABAergic PV/NKX2.1 and cholinergic neurons respectively. By clarifying the spatial molecular identity of GPe D2R neurons in the mouse, this work provides the basis for future studies aiming at disentangling the action of dopamine within the GPe.

## 1 INTRODUCTION

The basal ganglia, comprising the striatum, the globus pallidus (GP), the subthalamic nucleus (STN), and the substantia nigra (SN), are part of an anatomical system involved in motor control, goal-directed behaviors, and habit formation [1, 2]. More than a simple relay station, the external globus pallidus (GPe) serves as a key hub in the basal ganglia network [1]. Indeed, GPe projection neurons integrate information from the two inputs nuclei of the basal ganglia, the striatum, and STN, and send processed information back to all basal ganglia nuclei, including the striatum, the internal GP (GPi), STN, and SN, thereby optimizing the selection, initiation, and execution of actions [1].

In rodents, most of GPe neurons are GABAergic projection neurons (~95%) complemented by cholinergic neurons (~5%) [2]. GPe GABAergic neurons are segregated into at least two distinct subpopulations, the prototypic and arky-pallidal GPe neurons which can be identified by their electrophysiological and protein expression profiles, projections sites, and functions [3-5]. Due to the complete lack of interneurons within the GPe, the intrinsic regulation of information processing mainly relies on GABA released from collateral axons of GPe neurons [6, 7] but is also achieved by various neuromodulators among which dopamine [8-10].

Early anatomical studies indicate that most of the midbrain dopamine neurons arising from the substantia nigra pars compacta (SNc) and to a lesser extent from the ventral tegmental area (VTA) send collateral axons in the GPe [11-13]. Moreover, converging evidence indicate that dopamine release can be evoked in the GPe [14] and that local intrapallidal dopamine release can profoundly modulate GPe neural activity [9, 10, 15]. These effects are largely ascribed to the activation of dopamine D2 receptors (D2Rs) [9, 16, 17] found presynaptically at the striato-pallidal terminals [18] and postsynaptically [19, 20] in both parvalbumin-positive and -negative GPe neurons [21]. Finally, compelling evidence suggest that disrupted dopamine signaling leads to abnormal synchrony among GPe neurons and is causally linked to motor deficits [22-25].

However, all these evidence come from studies performed in rats leaving open the question of the distribution, the molecular and electrophysiological identity of D2R-expressing cells in the mouse GPe neurons. Given the increasing set of tools available to label, monitor, and manipulate cell-type GPe circuits in mice, a more comprehensive spatio molecular characterization of GPe D2R neurons is needed to better understand the role of dopamine D2R signaling among the different classes of GPe neurons. Here, we combined single molecule *in situ* hybridization, cell type-specific imaging analyses, and electrophysiology to characterize D2R-expressing cells in the mouse GPe. Our analysis revealed that GPe D2R neurons are not evenly distributed along the rostrocaudal and dorsoventral axis and indicate that GPe D2R neurons segregate in two subpopulations with distinct molecular and electrophysiological features. Overall, the present findings provide a useful resource to parse the role of D2R within the GPe.

## 2 MATERIALS AND METHODS

### 2.1 Animals

Male C57BL/6 (n = 3) from Charles River Laboratories, Drd2^Cre/+^ (n = 4), Drd2^Cre/+^; Ai9^f/+^ (n = 6) and Drd2^Cre/+^; Ribotag^f/+^ (n = 29) were used in the present study. Drd2^Cre/+^; Ribotag^f/+^ mice were generated as previously described [26]. Male 8- to 12-week-old mice (25-30 gr) were used in the current study. All mice were housed in groups of 2 to 5 per cage (standard sizes according to the European animal welfare guidelines 2010/63/EU) and maintained in a 12 h light/ dark cycle (lights on from 7:00 am to 7:00 pm), in stable conditions of temperature (22°C) and humidity (60%), with food and water provided *ad libitum*. All animal procedures were conducted following the guidelines of the French Agriculture and Forestry Ministry for handling animals (authorization number/license B34-172-41) and approved by the relevant local and national ethics committees (authorizations APAFIS#14875 and APAFIS#14255).

### 2.2 Stereotaxic Injection into the Ventral-posterior GPe

Surgeries were performed on 8-10 weeks old Drd2^Cre/+^; Ribotag^f/+^ and Drd2^Cre/+^ male mice. Animals were anesthetized with a mixture of ketamine (Imalgene 500, 50 mg/ml, Merial), 0.9% NaCl solution (weight/vol), and xylazine (Rompun 2%, 20 mg/ml, Bayer) (2:2:1, i.p., 0.1 ml/30 g) and mounted on a stereotaxic apparatus. The microinjection needle was connected to a 10 μl Hamilton syringe and filled with adeno-associated virus (AAV) containing ChR2-Td-Tomato, AAV2/1.CAGGS.flex.ChR2.tdTomato.SV40 (titer, 3.28E+12 GC/ml, Addgene #18917) (UPenn vector core, Philadelphia, USA) or AAV2/5.EF1a.DIO.eYFP (titer, 4E+12 GC/ml, Addgene #27056) (UNC vector core, Chapel Hill, USA). A microinjection needle was placed into the GPe (A/P = -1.5 mm; Lat. =2.5 mm; D/V = -3.75 mm) and a volume of 0.2 μl was injected over 5 min. The injector was left in place for an additional 5 min to allow for the diffusion of virus particles away from the injection site. Wounds of mice were sealed by suture. Animals were then returned to their home cages for a 14-day recovery period.

### 2.3 Drugs and Treatment

Quinpirole (1.0 mg/kg, i.p.) and raclopride (0.5 mg/kg) were purchased from Tocris and dissolved in 0.9% (w/v) NaCl (saline). Both drugs were administered at doses known to induce strongly increased pS32-cFos expression [27, 28]. Drd2^Cre/+^;Ribotag^f/+^ mice were habituated to handling and saline injection three consecutive days before the experiment. Drugs were administrated on day 4. Mice were perfused as described below 90 min after injection.

### 2.4 Tissue Preparation and Immunofluorescence

Tissue preparation and immunofluorescence were performed as previously described [29]. Mice were anaesthetized with Euthasol^®^ (360 mg/kg, i.p., TVM lab, France) and transcardially perfused with 4% (weight/vol) paraformaldehyde in 0.1 M sodium phosphate buffer (pH 7.5). Brains were post-fixed overnight in the same solution and stored at 4°C. Brains were sliced (30-µm thick sections) with a vibratome (Leica, France) and stored at -20°C in a solution containing 30% (*vol/vol*) ethylene glycol, 30% (vol/vol) glycerol and 0.1 M sodium phosphate buffer (PBS), until they were processed for immunofluorescence [30]. Coronal sections containing the ventral-posterior GPe were identified using a mouse brain atlas [31]. The immunofluorescence was performed as follow: *day 1*, free-floating sections were a) rinsed three times 10 min in PBS, b) incubated 15 min in 0.1% (*vol/vol*) Triton X-100 in PBS, c) rinsed again three times 10 min in PBS, d) blocked for 1 h in a solution of 3% BSA in PBS and e) incubated 72 hours at 4°C with the primary antibodies (Table **S1**) diluted in a PBS solution containing 1% BSA and 0.15% Triton X-100; *day 2*, sections a) were rinsed thrice for 10 min in PBS, b) incubated for 45 min with secondary antibodies with goat Cy2-, Cy3- and Cy5-coupled (1:400, Jackson Immunoresearch) and/or goat alexafluor 488 (1:400, Life Technologies) secondary antibodies and c) rinsed for 10 minutes (twice) in PBS before mounting in DPX (Sigma-Aldrich, Saint-Quentin Fallavier, France). Confocal microscopy was carried out at the Montpellier RIO Imaging Facility. Images covering the rostrocaudal extension of the GPe were acquired using NanoZoomer (Hamamatsu). Double-immunolabeled images were single confocal sections, acquired using sequential laser scanning confocal microscopy (Leica SP8). HA-positive cells were pseudocolored in green while other markers were pseudocolored in red. All parameters were held constant for all sections from the same experiment. Images used for quantification were all single confocal sections. HA-positive cells were manually counted in the ventral-posterior GPe (from bregma -1.00 to -1.56 mm). Adjacent serial sections were never counted for the same marker to avoid any potential double counting of hemisected neurons. Values in the histograms in Fig. (**1**) represent an estimated percentage of HA-positive neurons throughout the rostro-caudal extension of the GPe (16 hemispheres per mouse, n = 4 mice). Values in the histograms in Figs. (**4****-****6** and **S3**) represent the co-expression as a percentage of HA-positive neurons (green) and as a percentage of cells expressing the different markers tested (red) (7-10 hemispheres per mouse, n = 6 mice). The total numbers of HA- and marker-positive cells counted are indicated between parentheses.

### 2.5 Single-molecule Fluorescent *in situ* Hybridization

Analyses of *Drd2*, *Pvalb*, *Penk*, *Chat,* and *Slc18a3* mRNAs expression were performed using single-molecule fluorescent *in situ* hybridization (smFISH) [32]. Brains from 3 C57BL/6 male mice were rapidly extracted and snap-frozen on dry ice and stored at -80°C until use. Sixteen μm coronal sections GPe) were collected (from bregma -1.00 mm to -1.56 mm) directly onto Superfrost Plus slides (Fisherbrand). Probes for *Drd2* (ACDBio; Mm-drd2-C3, Cat# 406501-C3), *Pvalb* (ACDBio; Mm-pvalb-C2, Cat# 421931-C2), *Penk* (ACDBio; Mm-penk-C1, Cat# 318761), *Chat* (ACDBio; Mm-chat-C1, Cat# 408731) and *Slc18a3* (ACDBio; Mm-slc18a3-C1, Cat# 448771) were used with the RNAscope Fluorescent Multiplex Kit (ACDBio; Cat# 320850) according to manufacturer’s recommendations. After incubation with fluorescent-labeled probes, slides were counterstained with DAPI and mounted with ProLong Diamond Antifade mounting medium (Thermo Fisher scientific P36961). Confocal microscopy and image analyses were carried out at the Montpellier RIO imaging facility. Double- and triple-labeled images from the region of interest were single confocal sections captured using sequential laser scanning confocal microscopy (Leica SP8). Values in the histograms represent co-expression as a percentage of *Drd2*-expressing cells (green) and as a percentage of cells expressing the other markers tested (*Pvalb*, *Chat*, *Penk,* and *Slc18a3*) (3-4 images in the GPe per mouse, n = 3 mice).

### 2.6 *Ex vivo* Electrophysiology

#### 2.6.1 Slice Preparation

Brain slices containing the GPe were prepared as previously described [33]. Briefly, Drd2^Cre/+^ or Drd2^Cre/+^;Ai9^f/+^ mice were deeply anaesthetized with a mixture of ketamine/xylazine (100 mg/kg and 20 mg/kg, respectively). Then, a thoracotomy was performed to allow transcardial perfusion of a saturated (Carbogen: 95% O2/5% CO_2_) iced-cold modified ACSF (cutting solution) composed of 250 mM sucrose, 10 mM MgSO_4_·7H_2_O, 2.5 mM KCl, 1.25 mM NaH_2_PO_4_·H_2_O, 0.5 mM CaCl_2_·H_2_O, 1.3 mM MgCl_2_, 26 mM NaHCO_3_, and 10 mM D-glucose. After decapitation, the brain was quickly removed, glued on the stage of a vibratome (VT1200S, Leica microsystems), immerged in saturated iced-cold cutting solution, and cut into coronal (300-µm thick) sections. Slices were then incubated at 34°C for 1 h in a standard ACSF saturated by bubbling carbogen and containing 126 mM NaCl, 2.5 mM KCl, 1.25 mM NaH_2_PO_4_·H_2_O, 2 mM CaCl_2_·H_2_O, 2 mM MgSO_4_·7H_2_O, 26 mM NaHCO_3_, and 10 mM D-glucose, supplemented with 5 mM glutathione and 1 mM sodium pyruvate. They were maintained at room temperature in the same solution until recording.

#### 2.6.2 Electrophysiology

Whole-cell patch-clamp experiments were performed in a submersion recording chamber under an upright microscope (Ni-E workstation, Nikon). Slices were bathed in ACSF containing 126 mM NaCl, 3 mM KCl, 1.25 mM NaH_2_PO_4_·H_2_O, 1.6 mM CaCl_2_·H_2_O, 2 mM MgSO_4_·7H_2_O, 26 mM NaHCO_3_, and 10 mM D-glucose (pH: 7.4; Osmolarity: 310-315 mOsm). Caudal GPe neurons were visualized with infrared differential interference contrast and fluorescence microscopy (Spectra X light engine, Lumencor). D2R-positive GPe cells were identified either by the fluorescence of eYFP or tdTomato depending on the mouse line used for the experiment. Recording pipettes (5-7 MΩ) were prepared from borosilicate glass capillaries (GC150F-10; Harvard Apparatus) with a horizontal puller (Sutter Instrument, Model P-97). They were filled with an internal solution composed of 135 mM K-gluconate, 3.8 mM NaCl, 1 mM MgCl_2_·6H_2_O, 10 mM HEPES, 0.1 mM Na_4_EGTA, 0.4 mM Na_2_GTP, and 2 mM Mg_1.5_ATP (pH: 7.25; Osmolarity: 290-295 mOsm). Experiments were conducted using a Multiclamp 700B amplifier and a Digidata 1440 digitizer controlled by Clampex 10.3 (Molecular Devices) at 34°C. Data were acquired at 20 kHz and low-pass filtered at 4 kHz. Whole-cell patch clamp recordings were not corrected for junction potential which was 13mV with a K-gluconate-based solution. All the recordings were performed in current-clamp mode and in the presence of ionotropic glutamatergic and GABAergic receptor blockers. NMDA receptors were inhibited by 50 µM D-(-)-2-amino-5-phosphonopentanoic acid (APV), AMPA/kainate receptors by 20 µM 6,7-dinitroquinoxaline-2,3-dione (DNQX) and GABA_A_ receptors by 10 µM GABAzine (SR95531). Intrinsic properties of the recorded neurons were investigated using increasing current pulse injections (50 pA steps, ranging from -100 to 250 pA, 1 s duration). Chemicals and pharmacologic compounds were purchased from Sigma-Aldrich and Tocris, respectively.

#### 2.6.3 Analyses and Statistics

Neuronal intrinsic properties analyses were performed with Clampfit 10.3 and Origin 7. Principal component (PCA) and dendrogram analyses were performed using Prism 9 (GraphPad Software) and the XLSTAT plug-in of Excel software. Statistical analysis was performed with Prism 9 (GraphPad Software). Population data are presented as mean ± SEM. Unpaired data were compared using the Mann-Whitney U test (MW-U) test. Comparisons of F-I relationships were performed with a two-way repeated-measures ANOVA test followed by a Bonferroni test for multiple comparisons. Data were considered statistically significant for *P* values < 0.05 (* *p* < 0.05; n.s., not significant).

## 3 RESULTS

### 3.1 Distribution of GPe D2R Neurons Along the Rostrocaudal and Dorsoventral Axis

**Fig. (1):**
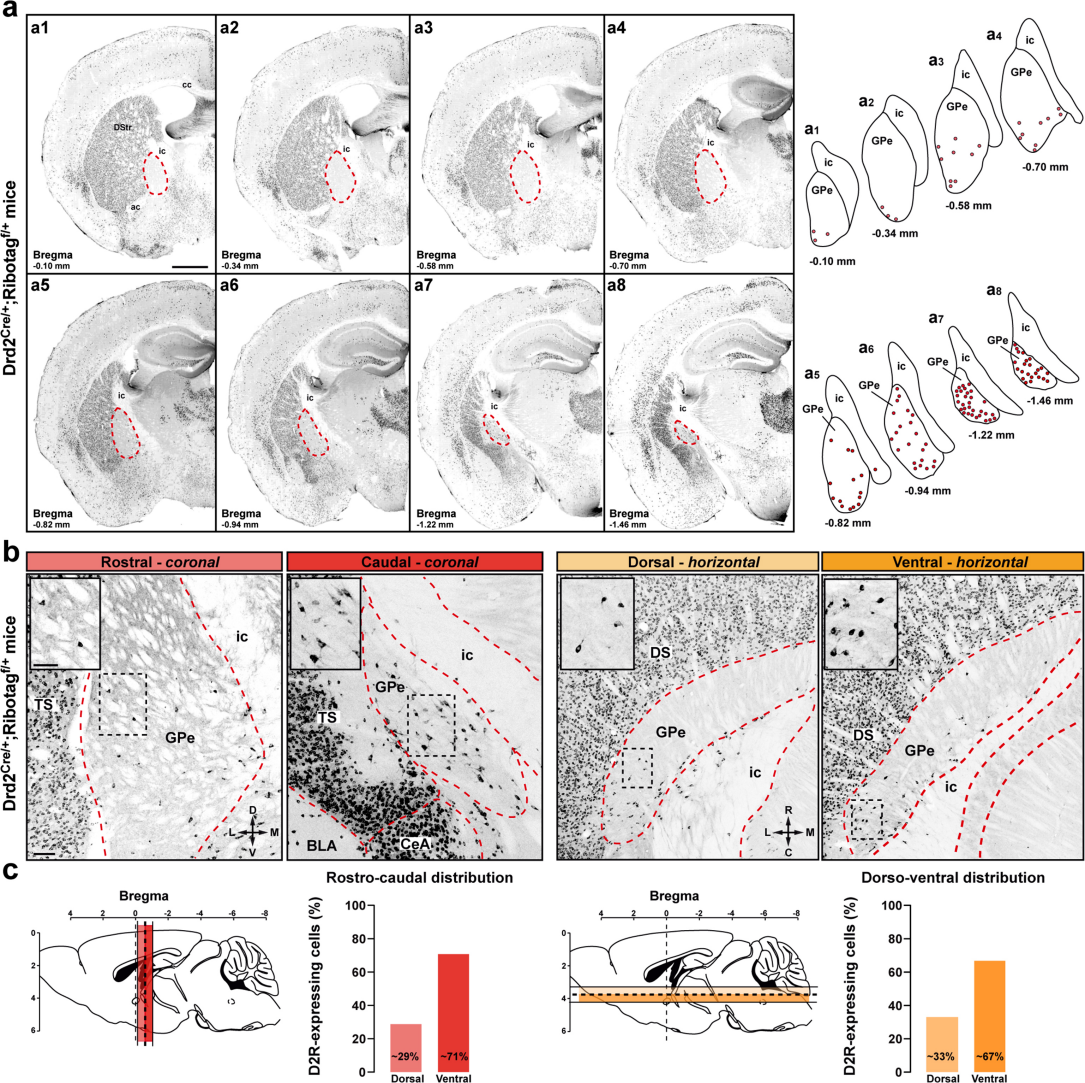
Distribution of D2R cells in the GPe of Drd2^Cre/+^;Ribotag^f/+^ mice. (**a**) Coronal GPe sections from Drd2^Cre/+^;Ribotag^f/+^ mice (n = 4) stained with HA. Representative distribution of HA-positive neurons across 8 coronal sections spanning throughout the rostrocaudal axis of the GPe (each dot representing a single neuron). Scale bar: 500 µm. (**b**), Representative HA immunolabeled images showing the rostrocaudal (left) and dorsoventral (right) distributions of HA-positive neurons in the GPe. Scale bar: 100 µm. Inserts are high-magnification images of areas delineated by the black stippled rectangle. Scale bar: 50 μm. (**c**) Histograms showing the % HA-positive cells in the GPe throughout the rostrocaudal (left) and dorsoventral (right) axis. Note the spatial distribution of D2R neurons is biased displaying the highest density in the ventral-posterior GPe. **Abbreviations:** DS: dorsal striatum; TS: the tail of the striatum; GPe: external globus pallidus; CeA: central amygdala; BLA: basolateral amygdala; ic: internal capsule; ac: anterior commissure.

Although early *in situ* hybridization studies revealed the presence of *Drd2* transcripts in the GPe [34], information regarding the distribution of D2R cells throughout the external globus pallidus (GPe) rostrocaudal axis are still lacking. Because dense GFP-labeled striatopallidal terminals detected in Drd2^eGFP/+^ mice preclude the identification of GPe D2R neurons [35, 36], we bred mice harboring the Drd2^Cre/+^ and the Ribotag^f/f^ [37] reporter allele expressing the ribosomal protein Rpl22 tagged with the hemagglutinin (HA) epitope in a Cre-dependent manner (Drd2^Cre/+^;Ribotag^f/+^ mice) [29]. The detection of HA immunoreactivity using anti-HA antibody revealed that GPe D2R cells found in the vicinity of TH-positive fibers (Fig. **S1**) were neurons and not microglia or glial cells as indicated by the lack of co-localization of HA with Iba1 and GFAP (Fig. **S2a**-**b**). Our analysis of antero-posterior and dorso-ventral sections revealed that GPe D2R neurons were not randomly distributed (Fig. **1a-b**). Indeed, the density of HA-positive gradually increased throughout the rostro-caudal axis being more concentrated in the ventro-posterior GPe (Fig. **1a**-**c**).

### 3.2 D2R Neurons Located in the Ventro-posterior GPe Project to Distinct Brain Areas

**Fig. (2):**
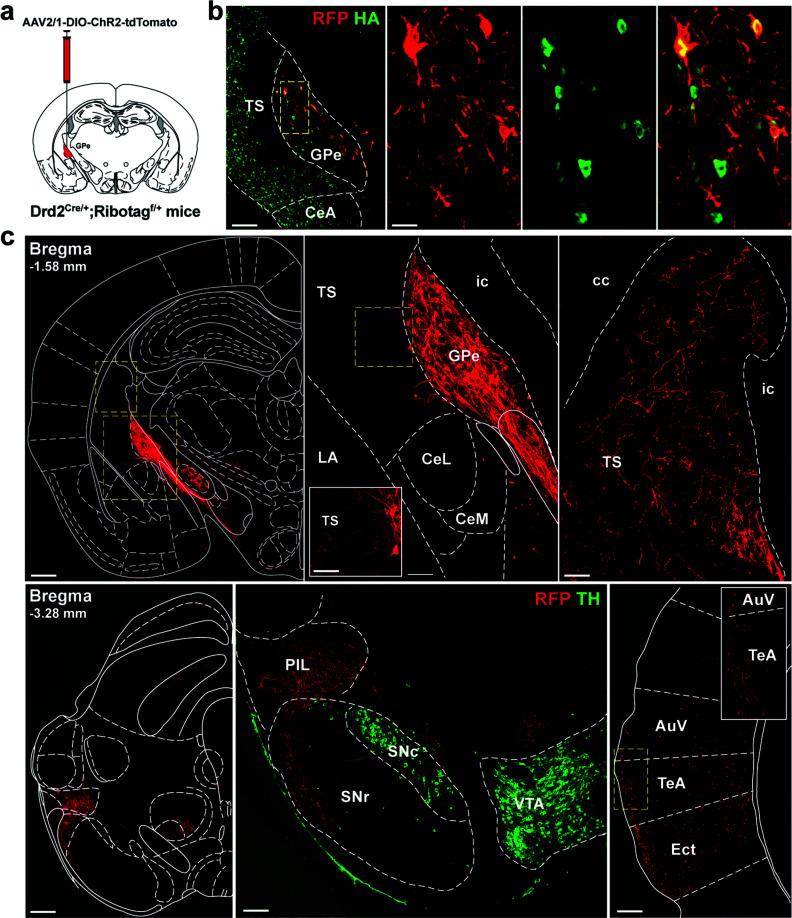
Identification of ventral-posterior GPe D2R neurons projecting areas. (**a**) Schematic representation of the site for the AAV2/1. CAGGS.flex.ChR2.tdTomato.SV40 injection into the caudal GPe of Drd2^Cre/+^;Ribotag^f/+^ mice. (**b**) Visualization of ChR2-expressing neurons in the GPe in Drd2^Cre/+^;Ribotag^f/+^ mice. Double immunofluorescence for mCherry (RFP) and HA (green). Scale bar: 70 µm. High magnification image of the area delineated by the yellow stippled rectangle. Scale bar: 20 µm. (**c**) Illustration of axon projection targets of ventral-posterior GPe D2R neurons. Note the clear identification of the pallido-striatal, pallido-nigral and pallido-cortical neurons. Immunofluorescence for tyrosine hydroxylase (TH, green) was used to identify SNc and VTA dopamine neurons. **Abbreviations:** TS: tail of the striatum; GPe: external globus pallidus; CeA: central amygdala; CeL: lateral part of the central amygdala; CeM: medial part of the central amygdala; BLA: basolateral amygdala; LA: lateral amygdala; PIL: posterior intralaminar nucleus; SNr: SN *pars reticulata*; SNc: SN *pars compacta*; VTA: ventral tegmental area; AuV: secondary auditory cortex, ventral area; TeA: temporal association cortex; Ect: ectorhinal association cortex; ic: internal capsule; cc: corpus callosum.

We then used an anterograde tracing strategy to identity the ventro-posterior GPe D2R neurons projecting areas (Fig. **2a**).We first expressed mCherry selectively in GPe D2R neurons of Drd2^Cre/+^; Ribotag^f/+^ mice to confirm that mCherry-labelled cells were found in HA-positive cells. As shown in Fig. (**2**), local injection into the caudal GPe triggered the expression of mCherry in HA-positive cells ruling out the possibility that the expression pattern of HA reflects the cumulative developmental expression history of the reporter gene (Fig. **2b**).

Subsequently, the areas containing visible mCherry-labelled axons were searched in the whole brain. Dense labelling was identified in several brain areas including the tail of the striatum (TS), the posterior intralaminar nucleus (PIL), the ventral part of the SN *pars reticulata* (SNr), the auditory cortex (AC), and the ectorhinal and temporal association cortices (TeA, Ect) (Fig. **2c**). The diversity of areas identified suggests that ventral-posterior GPe D2R neurons are composed of segregated neural populations comprising at least pallido-striatal, pallido-nigral, and pallido-cortical neurons. The absence of detectable fibers in the STN suggests also little overlap between GPe D2R and STN-projecting GPe neurons.

### 3.3 Electrophysiological Signatures of D2R Neurons Located in the Ventral-posterior GPe

**Fig. (3):**
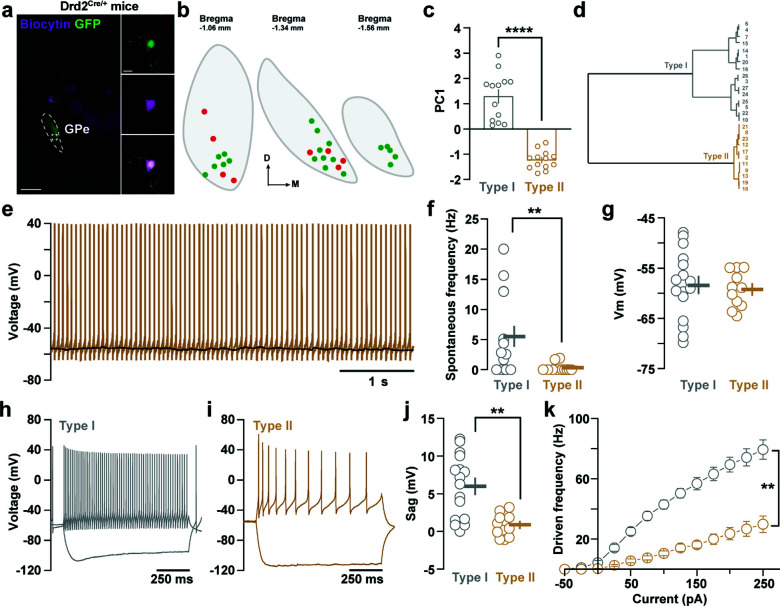
Electrophysiological signature of D2R-expressing neurons located in the ventral-posterior portion of the GPe. (**a**) Low magnification epifluorescent image of a coronal 300-µm thick section showing eYFP-expressing (green) and a biocytin-filled (magenta) GPe neurons. Insets on the left display the biocytin-filled D2R-positive GPe neuron at higher magnification. Scale bar: 10 μm. (**b**) Distribution on the rostro-caudal axis of the recorded cells. Green and red dots correspond to recordings performed in Drd2^Cre/+^; AAV-DIO-eYFP (n = 4 mice) and in Drd2^Cre/+^; Ai9^f/+^ (n = 6 mice) mice, respectively. (**c**) Graph showing the existence of two distinct neuronal populations by principal component analysis. (**d**) Dendrogram of two main D2R-expressing GPe neuron populations based on their electrophysiological properties. (**e**) Representative voltage trace of type II GPe neurons. (**f-g**) Graphs depicting spontaneous firing rate (**f**) and resting membrane potential (Vm) (**g**) of type I and type II GPe neurons. (**h-i**) Representative traces of voltage response to current step injection (-100 and +100 pA) for a type I (**h**) and a type II (**i**) GPe neuron. (**j**), Graph representing the sag value between type I and type II GPe neurons. (**k**) Frequency-current (**F-i**) curve showing the range of firing frequency of type I and type II GPe neurons.

We then looked for the characterization of the electrophysiological signatures of D2R GPe neurons using patch-clamp recordings in coronal brain slices obtained from Drd2^Cre/+^ mice transfected with an AAV-EF1a-DIO-eYFP (Fig. **3a**) or Drd2^Cre/+^;Ai9^f/+^ mice. To define the electrophysiological profile of these neurons, eYFP- or tdTomato-positive neurons were recorded in the presence in the ACSF of GABAergic and glutamatergic synaptic transmission blockers (GABAzine, 10 µM; DNQX, 20 µM and APV, 50 µM). 27 neurons located in the caudal portion of the GPe (Fig. **3b**) were recorded in current-clamp mode and several parameters such as resting membrane potential, spontaneous firing rate, driven firing rate, sag amplitude, or firing frequency accommodation were collected from each cell. Principal component analysis performed on all these parameters revealed the existence of two distinct cell types within D2R GPe neurons according to their electrophysiological signature (Fig. **3c-d**). Type I neurons (n = 13) were characterized by their higher spontaneous firing rate (Fig. **3e-g**), the presence of stronger sag (Fig. **3h-j**), and the capacity to fire at higher frequencies upon current injections (Fig. **3k**) compared to type II neurons (n = 14). These electrophysiological signatures were pretty similar to the properties of choline acetyltransferase (ChAT)-positive and ChAT-negative pallido-cortical GPe neurons described previously [38].

### 3.4 D2R Neurons Located in the Ventral-posterior GPe are Molecularly Distinct

**Fig. (4):**
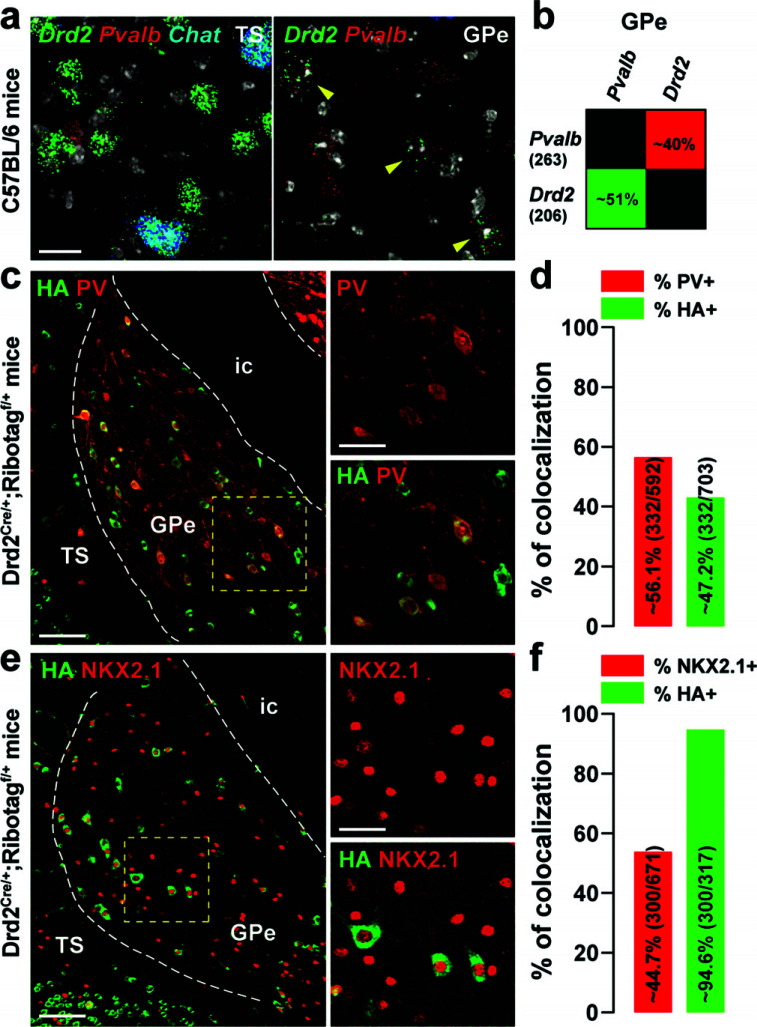
Distribution of *Drd2* among GPe PV and NKX2.1 neurons. (**a**) High magnification of confocal images of coronal brain section of the dorsal striatum (DS, left) and external globus pallidus (GPe, right) from C57BL/6 mouse (n = 3 mice) showing the distribution of *Drd2* (green), *Pvalb* (red) and *Chat* (blue, only for the DS) expression detected with single-molecular fluorescent *in situ* hybridization. Yellow arrows identified *Drd2*/*Pvalb* positive neurons. Slides were counterstained with DAPI (white). Scale bar: 10 μm. (**b**) Quantification of the overlap between neurons co-expressing *Drd2*, and *Pvalb* in the ventral-posterior GPe. Values in parentheses indicate the number of neurons identified for each marker (n = 3 mice). Percentages of co-labelling are represented in a matrix with probes in columns among neurons labeled with probes in rows (~51% of *Drd2*-positive neurons were also *Pvalb* positive. (**c**, **e**) Double immunofluorescence for HA (*green*) and parvalbumin (red, PV) (**c**) and NKX2.1 (red) (**e**) in the caudal GPe of Drd2^Cre/+^;Ribotag^f/+^ mice (n = 6 mice). Scale bar: 50 μm. High-magnification images of areas delineated by the yellow stippled squares. Scale bar: 20 μm. (**d**, **f**) Histograms showing the co-expression as a percentage of HA-labeled neurons (green, HA^+^) and as a percentage of cells expressing PV (red, PV^+^) (**d**) and NKX2.1 (red, NKX2.1^+^) (**f**). The numbers of HA^+^, PV^+^, and NKX2.1^+^ cells counted are indicated in parentheses. **Abbreviations:** TS: the tail of the striatum; GPe: external globus pallidus; ic: internal capsule.

**Fig. (5):**
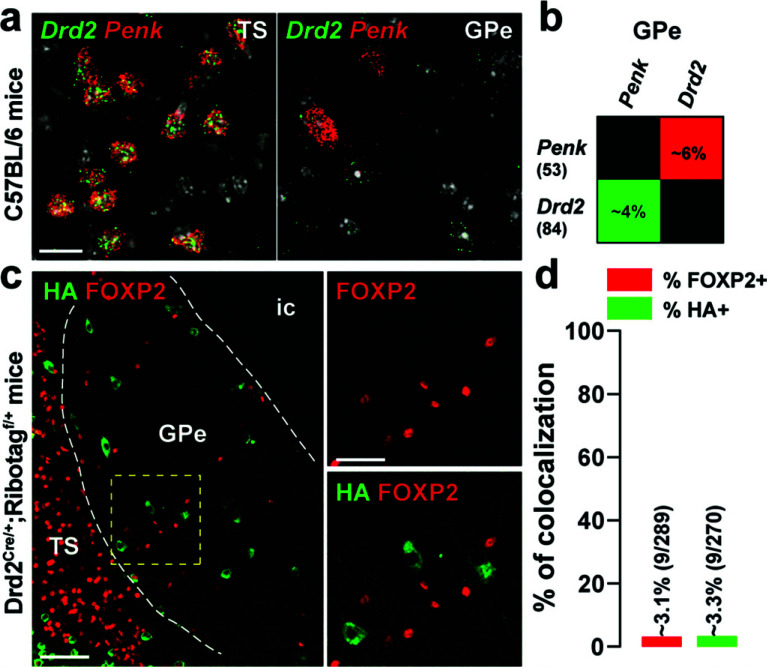
Distribution of *Drd2* among GPe *Penk* and FOXP2 neurons. (**a**) High magnification of confocal images of coronal brain section of the dorsal striatum (DS) and external globus pallidus (GPe) from C57BL/6 mouse (n = 3 mice) showing the distribution of *Drd2* (green) and *Penk* (red) expression detected with single-molecular fluorescent *in situ* hybridization. Slides were counterstained with DAPI (white). Scale bar: 10 μm. (**b**) Quantification of the overlap between neurons co-expressing *Drd2* and *Penk* in the ventral-posterior GPe as described in Fig. (**2b**). (**c**) Double immunofluorescence (*right panels*) for HA (*green*) and FOXP2 (red) in the caudal GPe of Drd2^Cre/+^; Ribotag^f/+^ mice (n = 6 mice). Scale bar: 50 μm. High-magnification images of areas delineated by the yellow stippled squares. Scale bar: 20 μm. (**d**) Histograms showing the co-expression as a percentage of HA-labeled neurons (green, HA^+^) and as a percentage of cells expressing FOXP2 (red, FOXP2^+^). The numbers of HA^+^ and FOXP2^+^ cells counted are indicated in parentheses. TS: the tail of the striatum; GPe: external globus pallidus; ic: internal capsule.

GABAergic neurons account for about 95% of the GPe neurons [1]. These neurons fall into two molecularly distinct intermingled neuronal populations identified by the expression of *Pvalb/Nkx2.1* and *Foxp2/Penk* transcripts [3, 4]. We, therefore, examined the relationship between these two populations and GPe D2R neurons. Using single-molecule fluorescent *in situ* hybridization (smFISH), we found that ~50% of *Drd2*-positive cells of the ventral-posterior GPe were *Pvalb* (Fig. **4a-b**). A similar proportion was observed when the percentage of immunofluorescence HA-positive neurons co-expressing parvalbumin (PV) was analyzed (Fig. **4c-d**). Consistent with the compartmentalization of PV-positive neurons in CB-lacking zones, less than 1% HA-expressing neurons were calbindin-28kD (CB) positive cells (Fig. **S3a**-**b**) [39]. We also found that a majority of HA-immunoreactive neurons expressed the transcription factor NKX2.1 (~94.6%) (Fig. **4e-f**). In contrast, our analysis revealed that less than ~5% of *Drd2*- or HA-positive neurons were found to be *Penk*- (Fig. **5a-b**) or FOXP2-expressing cells (Fig. **5c-d**).

**Fig. (6):**
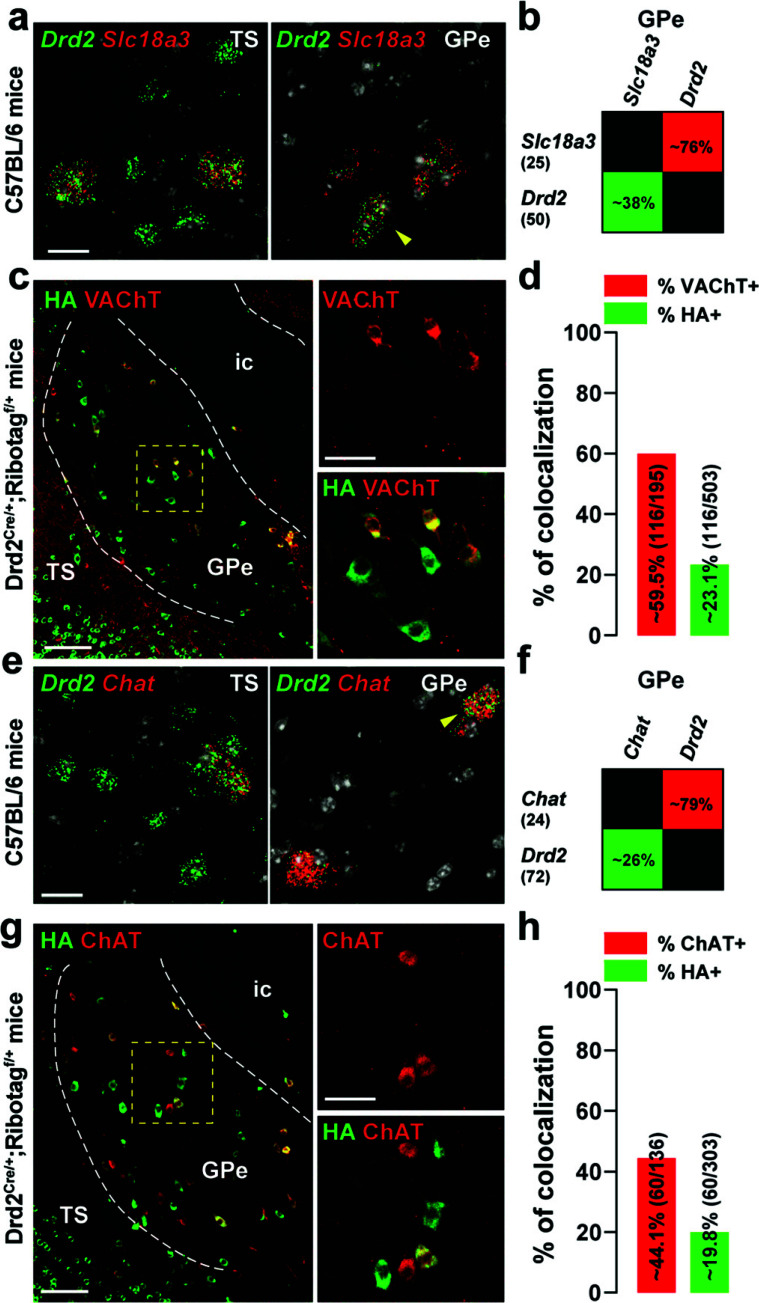
Distribution of D2R among GPe cholinergic neurons. (**a**, **e**) High magnification of confocal images of coronal brain section of the dorsal striatum (DS) and external globus pallidus (GPe) from C57BL/6 mouse (n = 3 mice) showing the distribution of *Drd2* (green), *Slc18a3* (red) (**a**) and *Chat* (red) (**e**) expression detected with single-molecular fluorescent *in situ* hybridization. Yellow arrows identified *Drd2*/*Slc18a3* and *Drd2*/*Chat* positive neurons in the DS and GPe. Slides were counterstained with DAPI (white). Scale bar: 10 μm. (**b**, **f**) Quantification of the overlap between neurons co-expressing *Drd2* and *Slc18a3* (**b**) or *Chat* (**f**) in the ventral-posterior GPe as described in Fig. (**2b**). (**c**, **g**) Double immunofluorescence (*right panels*) for HA (*green*), VAChT (red) (**c**) and ChAT (red) (**g**) in the caudal GPe of Drd2^Cre/+^;Ribotag^f/+^ mice (n = 5 mice). Scale bar: 50 μm. High-magnification images of areas delineated by the yellow stippled squares. Scale bar: 30 μm. (**d**, **h**) Histograms showing the co-expression as a percentage of HA-labeled neurons (green, HA^+^) and as a percentage of cells expressing VAChT (red, VAChT^+^) (**d**) or ChAT (red, ChAT^+^) (**h**). The numbers of HA^+^, VAChT^+^_,_ and ChAT^+^ cells counted are indicated in parentheses. TS: the tail of the striatum; GPe: external globus pallidus; ic: internal capsule.

The caudal GPe also contains a substantial number of cholinergic neurons which represent 5% of GPe neurons [1, 40, 41]. Therefore, the degree of co-localization of *Drd2*- or HA-labeled cells with *Slc18a3* and Chat encoding the vesicular acetylcholine transporter (VAChT) and the choline acetyltransferase (ChAT) was assessed respectively (Fig. **6**). Our smFISH analysis revealed that *Drd2*-positive neurons of the ventral-posterior GPe co-expressed *Slc18a3* (~38%) and *Chat* (~26%) (Fig. **6a-b, e-f**), an observation confirmed by immunofluorescence using VACHT and ChAT antibodies (Fig. **6c-d, g-h**). Altogether, our results indicate that in the caudal GPe, D2R is preferentially expressed by PV/NKX2.1 neurons and to a lesser extent by cholinergic neurons.

## 4 DISCUSSION

Since their development more than a decade ago, Drd2^eGFP/+^ or Drd2^Cre/+^ mice crossed with mouse reporter lines expressing any other fluorescent proteins (eGFP, tdTomato) or epitope-tagged proteins (Ribotag) have made it possible with great precision the distribution of D2R-expressing cells in various brain areas. If such tools allowed refining the anatomical distribution and cellular composition of D2R neurons within the striatum [36, 42], it also largely contributed to the map distribution of D2R-expressing cells in brain areas where D2R promotor activity is low such as the hippocampus [29, 43, 44], cerebral cortex [33, 45] or cerebellum [46]. By using such approaches and analyzing the distribution of HA-immunoreactive cells in Drd2^Cre/+^;Ribotag^f/+^ mice [29], we found that GPe D2R neurons comprise pallido-striatal, pallido-nigral, and pallido-cortical neurons preferentially localized in the caudal portion of GPe. Our molecular and electrophysiological characterization also unveiled that GPe D2R neurons fall into two distinct neuronal populations, the GABAergic PV/NKX2.1 neurons, and cholinergic neurons.

The detection of low density of *Drd2* mRNA throughout the GPe was first evidenced by *in situ* hybridization studies performed in rats [21, 47-49]. While a widespread expression has been initially reported in the rat [21], mouse GPe *Drd2*-expressing cells are mainly detected in the ventral-posterior part as revealed by the expression pattern of HA-positive cells throughout the antero-posterior GPe axis in Drd2^Cre/+^; Ribotag^f/+^ mice. The analysis of mCherry-labelled axons of Drd2^Cre/+^; Ribotag^f/+^ mice injected with AAV2/1. CAGGS. flex.ChR2.tdTomato.SV40 in the caudal GPe allowed us to determine that GPe D2R neurons comprise pallido-striatal, pallido-nigral, pallido-cortical but not pallido-subthalamic neurons. These data are reminiscent of those obtained with previous anterograde tracing studies performed in rats [40, 50]. Interestingly, similar to the TS [51], caudal GPe neurons are highly connected with brain areas involved in visuomotor and auditory processing suggesting that caudal GPe neurons may preferentially regulate sensory-related information.

The vast majority of GPe neurons are GABAergic projection neurons displaying an important level of molecular and functional heterogeneity [3, 4, 52-55]. Among the distinct classes of GPe neurons, *Drd2*/HA-positive neurons strongly co-express markers of GPe prototypic neurons (*Pvalb*/PV and NKX2.1) but lack those identifying GPe arkypallidal neurons (*Penk* and FOXP2) [1]. This latter observation contrasts with previous results obtained in rats, in which *Drd2* mRNA expression partially overlaps with GPe *Penk*-positive neurons [21] suggesting that the distribution and molecular identity might not be strictly conserved between mice and rats. Such differences might explain why the increased number of cFos-positive cells detected in the rat GPe following the activation or the blockade of D2R [34, 56, 57] was not observed in mice (Fig. **S4**) but could also account for the distinct motor responses induced by quinpirole. Thus, while systemic quinpirole administration causes a biphasic motor response characterized by a transient hypolocomotion followed by enhanced activity in rats [58, 59], only sustained reduced locomotion is observed in mice [27].

NKX2.1 also identifies cholinergic neurons that are primarily located in the caudal GPe [1, 41, 60]. Our analysis unveiling that ventral-posterior GPe *Drd2*/HA neurons co-expressed *Slc18a3*/VAChT and *Chat*/ChAT, indicates that in addition to being expressed in GPe prototypic neurons, *Drd2*/D2R are also present in a fraction of GPe cholinergic neurons. Interestingly, our electrophysiological recordings also revealed two distinct neuronal populations according to cluster analysis. Type I caudal GPe neurons were characterized by hyperpolarization-activated cation currents (Ih), and higher spontaneous and maximal firing rates which is reminiscent of the features of GABAergic PV/NKX2.1 neurons [4, 38, 54]. Conversely, type II GPe neurons had lower spontaneous and maximal firing rates and almost no Ih current which is consistent with the electrophysiological profile of cholinergic neurons [38]. Finally, our results are in line with the previous tract-tracing studies indicating that caudal GPe PV neurons largely project to the TS, PIL, and SNr pars lateralis but not the STN [50] while caudal GPe ChAT neurons innervate the auditory cortices [40].

Among the basal ganglia nuclei, the GPe is the one displaying the highest density of glial cells namely astrocytes [61]. Interestingly, recent studies indicate that dopamine regulates GPe astrocytes functions. Thus, the application of quinpirole reduces GPe astrocytic spontaneous Ca^2+^ transients through a mechanism requiring the activation of D3R but not D2R [61]. Moreover, D2R activation has been shown to regulate the activity of the glial transporter (GAT-3) facilitating GABA uptake by GPe astrocytes [62]. Finally, although recent evidence unveil the presence of functional D2R in GPe astrocytes [63], the lack of detection of GFAP/HA-positive cells in the GPe suggests that, if any, basal D2R expression in GPe astrocytes is low.

## CONCLUSION

To summarize, our work provides the first spatiomolecular characterization of D2R-expressing cells in the mouse GPe. Future work will be necessary to disentangle the functional role of D2R within the GABAergic PV/NKX2.1 and cholinergic neurons of the caudal GPe.

## Data Availability

The data supporting the findings of this study are available within the paper and its supplementary materials files or available from the corresponding author upon reasonable request.

## LIST OF ABBREVIATIONS

AC: Auditory Cortex
GP: Globus Pallidus
PIL: Posterior Intralaminar Nucleus
SN: Substantia Nigra
STN: Subthalamic Nucleus
