# Supplementary material for: Spatiomolecular Characterization of Dopamine D2 Receptors Cells in the Mouse External Globus Pallidus
Source: Curr Neuropharmacol. 2023 Jul 31;22(9):1528–39. doi: 10.2174/1570159X21666230720121027 (PMC11097984; doi:10.2174/1570159X21666230720121027)
Supplement: Supplementary file 1 [file CN-22-1528_SD1.pdf]

## Supplementary Material

# Spatiomolecular Characterization of Dopamine D2 Receptors Cells in the Mouse External Globus Pallidus

Julie Espallergues[1], Jihane Boubaker-Vitre[1], Audrey Mignon[1], Maelle Avrillon[1], Morgane Le Bon-Jego[2], Jerome Baufreton[2] and Emmanuel Valjent[1,*]

*[1]IGF, University Montpellier, CNRS, Inserm, F-34094 Montpellier, France; [2]University Bordeaux, CNRS, IMN, UMR 5293, F-33000 Bordeaux, France*

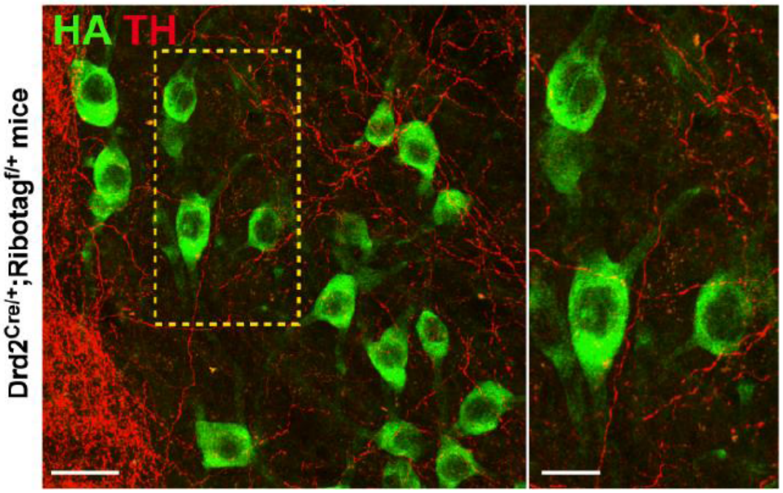


**Fig. (S1).** TH-positive fibers in the ventro-posterior GPe in Drd2$^{Cre/+}$;Ribotag$^{f/+}$ mice. HA (green) and tyrosine hydroxylase (red, TH) immunofluorescence in the ventro-posterior GPe of Drd2$^{Cre/+}$;Ribotag$^{f/+}$ mice. Scale bar, 20 μm. Insert is a high magnification image of areas delineated by the yellow stippled rectangle and show TH-positive fibers that made appositions with nearby HA-expressing cells. Scale bar, 10 μm.

1875-6190/24 $65.00+.00 © 2024 Bentham Science Publishers

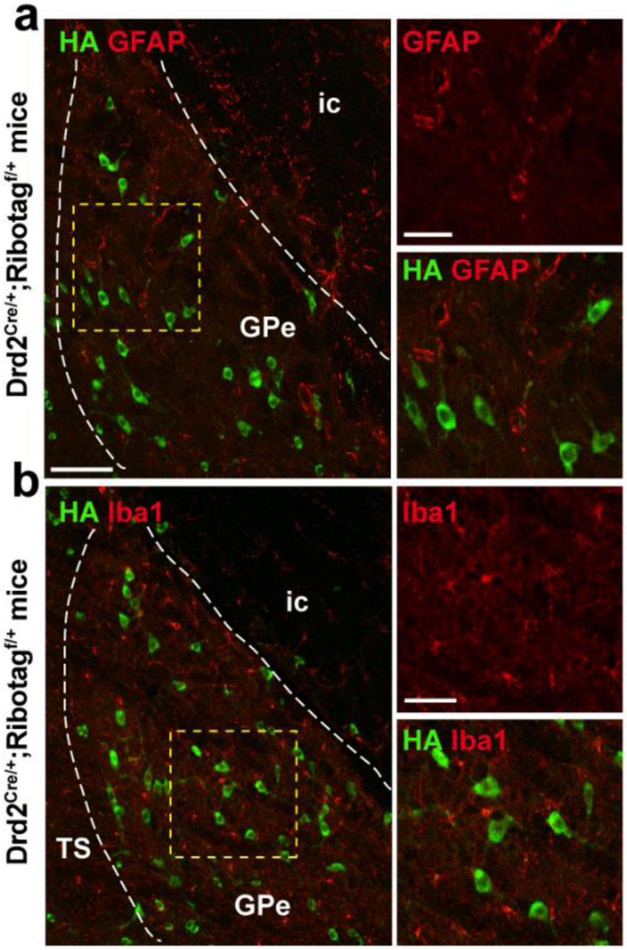


**Fig. (S2).** GPe D2R cells are not microglial or glial cells. (**a**, **b**) Double immunofluorescence for HA (green) and Iba1 (red) (**a**) or GFAP (red) (**b**) in the caudal GPe of Drd2$^{Cre/+}$;Ribotag$^{f/+}$ mice (n = 4 mice). Scale bar: 50 µm. High magnification images of areas delineated by the yellow stippled squares. Scale bar: 30 µm. No colocalization was found between HA-positive cells and Iba1 or GFAP labeled cells. TS: tail of the striatum; GPe: external globus pallidus; ic: internal capsule.

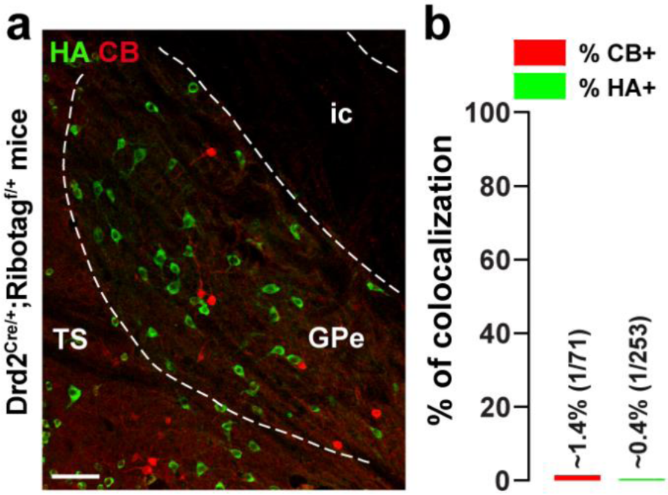


**Fig. (S3).** Distribution of D2R neurons among calbindin-D28k-positive neurons. (**a**) Double immunofluorescence for HA (green) and calbindin-28kD (red, CB) in the caudal GPe of Drd2$^{Cre/+}$;Ribotag$^{f/+}$ mice (n = 4 mice). Scale bar: 50 µm. (**b**) Histograms showing the co-expression as percentage of HA-labeled neurons (green, HA$^{+}$) and as percentage of cells expressing CB (red, CB$^{+}$). Numbers of HA+ and CB$^{+}$ cells counted are indicated in parentheses. TS: tail of the striatum; GPe: external globus pallidus; ic: internal capsule.

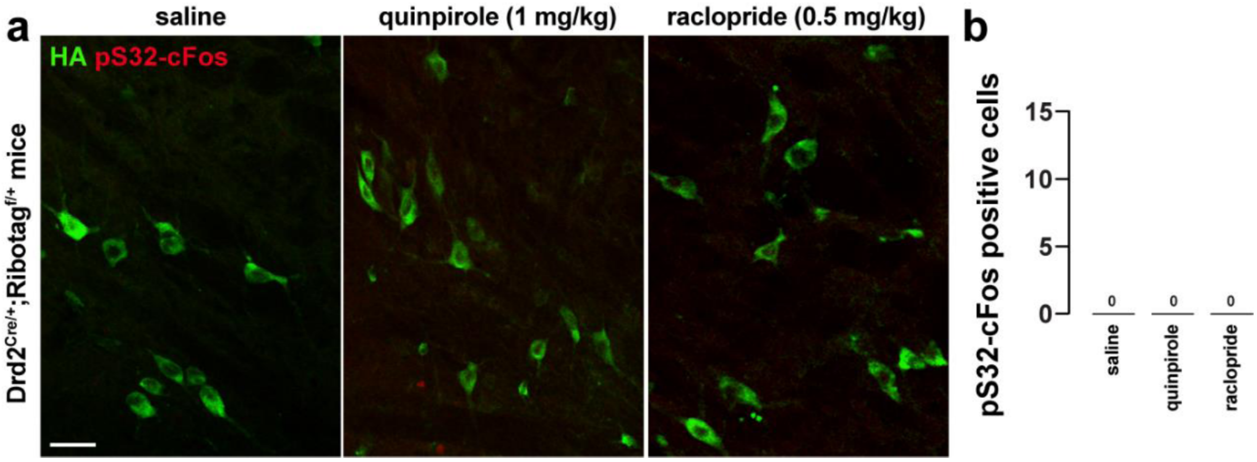


**Fig. (S4).** Quinpirole and raclopride fail to induce pS32-cFos in GPe D2R cells. (**a**) Double immunofluorescence for HA (green) and pS32-cFos (red) in the caudal GPe of $Drd2^{Cre/+}$;$Ribotag^{f/+}$ mice after saline, quinpirole (1 mg/kg) or raclopride (0.5 mg/kg) administration. Scale bar: 20 µm. (**b**) Quantification of pS32-cFos immunoreactive neurons in the caudal GPe of $Drd2^{Cre/+}$;$Ribotag^{f/+}$ mice treated with saline (n = 3 mice), quinpirole (1 mg/kg) (n = 3 mice) or raclopride (0.5 mg/kg) (n = 3 mice) and perfused 90 min after injection. Note the lack of detection of pS32-cFos immunoreactive cells in the GPe.

**Supplemental Table 1: List of primary antibodies**

| Antigen | Host | Dilution | Supplier | Catalog no |
|---|---|---|---|---|
| GFAP | Rabbit | 1:2000 | Dako | Z0334 |
| Iba1 | Rabbit | 1:1000 | Wako | #019-19741 |
| HA | Rat | 1:500 | Sigma | 12158167001 |
| HA | Rabbit | 1:1000 | Rockland | 600-401-384 |
| HA | Mouse | 1:1000 | Covance | MMS101R |
| RFP | Rabbit | 1:1000 | MBL | PM005 |
| TH | Rabbit | 1:1000 | Millipore | #AB152 |
| pS32-cFos | Rabbit | 1:500 | Cell Signaling | #11919 |
| Calbindin-D28k | Rabbit | 1:1000 | Swant | CB382 |
| Parvalbumin | Rabbit | 1:1000 | Swant | PV25 |
| VAChT | Rabbit | 1:1000 | Synaptic System | #139103 |
| ChAT | Goat | 1:500 | Millipore | #AB144 |
| FOXP2 | Rabbit | 1:500 | Abcam | #ab16046 |
| NKX2.1 | Rabbit | 1:500 | Santa Cruz | sc-13040 |
